# The effects of acute angle closure crisis on corneal endothelial cells in patients with type 2 diabetes mellitus

**DOI:** 10.3389/fendo.2022.956780

**Published:** 2022-08-30

**Authors:** Lin Cong, Xiaojing Pan, Yiping Xia, Yangyang Zhang, Jun Cheng, Yanling Dong

**Affiliations:** ^1^ Eye Institute of Shandong First Medical University, Qingdao Eye Hospital of Shandong First Medical University, Qingdao, China; ^2^ State Key Laboratory Cultivation Base, Shandong Provincial Key Laboratory of Ophthalmology, Qingdao, China; ^3^ School of Ophthalmology, Shandong First Medical University, Qingdao, China; ^4^ Qingdao Traditional Chinese Medicine Hospital (Qingdao Hiser Hospital), Qingdao, China

**Keywords:** acute angle closure crisis, diabetic keratopathy, type 2 diabetes mellitus, cornea endothelial cells, intraocular hypertension

## Abstract

**Objective:**

This study investigated the effects of acute angle closure crisis (AACC) on the corneal endothelial cells in patients with type 2 diabetes mellitus (DM) to identify the factors that cause corneal endothelial cell injury.

**Methods:**

We examined 154 patients who visited Qingdao Eye Hospital for AACC in one eye (154 eyes; 28 men and 126 women; mean age of 68 ± 8 years). We divided the participants into non-DM, DM well-control, and DM poor-control groups, with the unaffected eyes used as controls. Each participant was evaluated at the hospital while under AACC. We measured the relevant index and corneal parameters of the participants for statistical analysis.

**Results:**

There were significant statistical differences in corneal parameters among the three groups. The decreased levels of central endothelial cell density (CD) and the percentage of hexagonal cells (6A) were statistically relevant among the groups (P<0.05). The AACC duration was correlated with CD loss rate among the groups (P<0.05). The DM duration was correlated with CD loss rate in the DM well-control group. Compared with the non-DM group, the level of 6A decreased more significantly in the DM group after AACC (P<0.05). The AACC duration in the DM well-control group was significantly shorter than in the non-DM and DM poor-control groups (P<0.001). The DM poor-control group showed significantly worse visual acuity when compared with the other groups (P<0.05).

**Conclusions:**

DM may impact the functional status of corneal endothelial cells. AACC can worsen the corneal endothelium damage in patients with DM. Blood glucose levels and the duration of intraocular hypertension are closely related to the severity of corneal endothelial injury.

## Introduction

Glaucoma is an irreversible eye disease that progressively damages the optic nerve. This typically results in vision loss and may even cause blindness, which seriously affects the quality of life. Acute angle closure crisis (AACC), which is also known as acute angle closure glaucoma, is an ophthalmic emergency with a high incidence rate in middle-aged and elderly persons. The clinical manifestations of AACC include sudden ophthalmodynia, headache, and nausea, which can easily be misdiagnosed as various neurological diseases, thus delaying treatment (1). A sharp increase in intraocular pressure over a short period can not only lead to progressive retinal ganglion cell apoptosis and characteristic optic nerve damage (2), but may also cause irreversible damage to corneal endothelial cells leading to corneal endothelial dysfunction (3–5).

Diabetes mellitus (DM) is a chronic metabolic disease that is often associated with peripheral nerve, cardia-cerebrovascular, and organ complications (6). As a relatively widespread condition, it is connected to a series of public health and economic problems (7, 8). In China, where the current study was conducted, the incidence of DM continues to increase on a yearly basis (9). Meanwhile, long-term poor glycemic control, such as that associated with DM, may also lead to secondary ocular complications, including diabetic keratopathy, diabetic cataract, and diabetic retinopathy (10). The main manifestations of diabetic keratopathy include decreased corneal nerve sensitivity, corneal epithelial injury, and abnormal corneal endothelial cell function. However, the pathogenesis of diabetic keratopathy remains unclear (11–14).

Pont et al. retrospectively analyzed differences in corneal endothelium between patients with and without DM with an additional focus on how cataract surgery influences corneal endothelium in patients with DM (14). Durukan investigated the connection between endothelium status and retinopathy severity in patients with DM (15), and Rim et al. considered patients with DM more likely to develop open angle glaucoma (16), though the relationship between DM and open angle glaucoma development remains controversial (17). However, there is still a lack of clinical evidence on corneal endothelial injury in patients with DM who have undergone AACC. Further, the relationship between glycemic control and the health of the corneal endothelium remains unclear.

To address this gap, this study analyzed corneal parameters in DM and non-DM patients who were treated for AACC at Qingdao Eye Hospital, thus evaluating the different effects of duration time, glycemic control, and intraocular pressure (IOP) on corneal endothelial injury. We also investigated the relationship between blood glucose levels and the degree of corneal endothelial injury in DM patients with acute glaucoma attack.

## Materials and methods

Using a retrospective study design, we consecutively collected and calculated AACC patients who visited Qingdao Eye Hospital (affiliated with Shandong First Medical University) between 2017 and 2020. AACC was diagnosed according to the typical ocular syndrome and accessory examination (18), at least two symptoms (vision blurring, scieropia/irisopsia, ocular pain, nausea/vomiting) and at least three ocular abnormal signs (corneal edema, gonioscopic confirmation of angle closure, mid dilated vertical oval pupil, glaucomatous fleck) (19). None of these participants had previously undergone binocular surgery, nor both eyes were affected by other corneal diseases that could affect IOP measurement. The patients had not received any eye examinations before the AACC attack. Because if those patients had received regular ocular examination, peripheral iridotomies could have been performed to avoid the AACC attack. For the lack of the data before AACC, we strictly filtrated the participants and used the corneal parameters of the other unaffected eye as control (20). Each participant was only affected by AACC in one eye, the other eye was in the pre-clinical stage. In each case, the unaffected eye had no glaucoma history, normal IOP, and a cup-to-disc ratio of less than 0.5. Ethics committee approval was obtained from the Ethics Review Board at Qingdao Eye Hospital. All study procedures adhered to the Declaration of Helsinki.

Once the participants got a confirmed AACC diagnosis upon visiting the clinic, they were immediately admitted to the inpatient department and given medical treatment to control IOP. Their medical history, best-corrected visual acuity (BCVA), IOP, HbA1c, and liver and renal function were obtained when they were admitted. Those without liver or kidney problems were given a 20% mannitol intravenous drip, while those with severe ocular inflammation were given a 10 mg dexamethasone intravenous infusion. Eye drops were also applied to reduce the inflammatory response and decrease IOP. Participants with IOP higher than 50 mmHg were treated with anterior chamber paracentesis to avoid optic nerve injury. The corneal limbal paracentesis cannot damage the central endothelium. For each participant, the following corneal parameters of both eyes were subjected to standardized ophthalmic examinations once IOP was well controlled (under 21 mmHg)—when corneal edema had disappeared, corneal transparency had recovered, and inflammatory reactions had completely faded. Those patients with uncontrolled IOP were excluded from the trial to receive surgical intervention. The control eyes were not prophylactic treated before examination. To minimize the error rate, the same experienced ophthalmic technician performed all the examinations using fixed equipment.

BCVA was recorded on a standard Snellen chart. IOP was measured using a Goldman Applanation Tonometer (AT-900 Haag-Streit, Swiss). A non-contact specular microscope (NSP-9900II KONAN, Japan) was used to detect central endothelial cell density (CD), the percentage of hexagonal cells (6A), maximum cell area (MAX), minimum cell area (MIN), average cell area (AVE), standard deviation of average area (SD) of AVE, and coefficient of variation of average area (CV) of AVE. In some participants, the nasal, temporal, superior, and inferior sites of the cornea were also detected. Central corneal thickness (CCT) was measured three times using an ultrasonic pachymeter (24-4400 Accutomw, USA), with the average of all such readings recorded for each participant. Data from the medical history (age, AACC duration time, and diabetes duration time) were also obtained for further analysis. In our case, the duration time of AACC referred to the onset of ocular discomfort. Although the IOP level during the early stage may not be as high as the onset of AACC, it may also affect corneal endothelial cells. Based on their histories with type 2 DM, we divided the participants into non-DM and DM groups. Based on their glycosylated hemoglobin (HbA1c), we subdivided the participants in the DM group into groups for DM well-control (HbA1c<7%) and DM poor-control (HbA1c≥7%) (21, 22) (**Table 1**).

**Table 1:** Demographic and ophthalmic characteristics of the study groups.

	Non-DM Group	DM Group		P value
		DM well-control	DM poor-control	
Cases (eyes)	67	39	48	
Gender (Male/Female)	11 / 56	6 / 33	11 / 37	
Age (years)	66 ± 8	71 ± 6	70 ± 7	
IOP (mmHg)	30.66 ± 16.70	30.33 ± 16.51	32.23 ± 14.93	P = 0.981^a^, P = 0.607^b^, P = 0.586^c^
HbA1c (%)	4.87 ± 0.54	6.04 ± 0.65	8.34 ± 1.91	**P < 0.001^a,b,c^**
AACC duration (days)	8.70 ± 5.66	5.77 ± 4.95	8.90 ± 5.64	**P = 0.009^a,c^**, P = 0.847^b^
Diabetes duration (years)	–	9.19 ± 6.31	9.78 ± 5.49	P = 0.659^c^
CD loss rate (%)	17.1	10.1	19.1	P = 0.114^a^, P = 0.593^b^, **P = 0.042^c^**
6A decline rate (%)	5.6	13.1	16.2	P = 0.050^a^, **P = 0.016^b^**, P = 0.765^c^

DM, diabetes mellitus; IOP, intraocular pressure; HbA1c, glycosylated hemoglobin; AACC, acute angle closure crisis; CD, cell density; 6A, percentage of hexagonal cell.

Bold values indicate statistically significant. (P < 0.05).

^a^Statistically significant difference between the non-DM group and DM well-control group.

^b^Statistically significant difference between the non-DM group and DM poor-control group.

^c^Statistically significant difference between the DM well-control group and DM poor-control group.

We conducted the statistical analyses using IBM SPSS (version 21.0 IBM, USA). In this study, the descriptive data are presented as means ± standard deviations. We used a paired t-test to compare the AACC eyes with the control eyes in each group. Next, we compared differences of corneal parameters between the non-DM, DM well-control, and DM poor-control groups using the one-way analysis of variance (ANOVA). We analyzed the correlations between age, IOP, HbA1c, AACC duration time, and diabetes duration time with CD loss rate and 6A decline rate using multivariate regression analyses. Finally, we used the chi-square test to analyze the categorical variables of visual acuity in multiple groups. Any differences were considered statistically significant at a probability level of *P*<0.05.

## Results

In total, we examined 154 participants (154 eyes with AACC), including 28 men and 126 women (mean age of 68 ± 8 years). For the eyes with AACC, average IOP was 46.14 ± 15.57 mmHg, with an AACC duration time of 8.68 ± 7.21 days (shortest being 1.5 hours and longest being 21 days). Other data about case number, gender, age, IOP, HbA1c, AACC duration, diabetes duration, CD loss rate, and 6A decline rate in each group was recorded and analyzed (**Table 1**). To clarify, two patients in the DM well-control group and seven patients in the DM poor-control group were diagnosed with DM at the time of visit. They were excluded from the statistics of diabetes duration.

**Figure 1:**
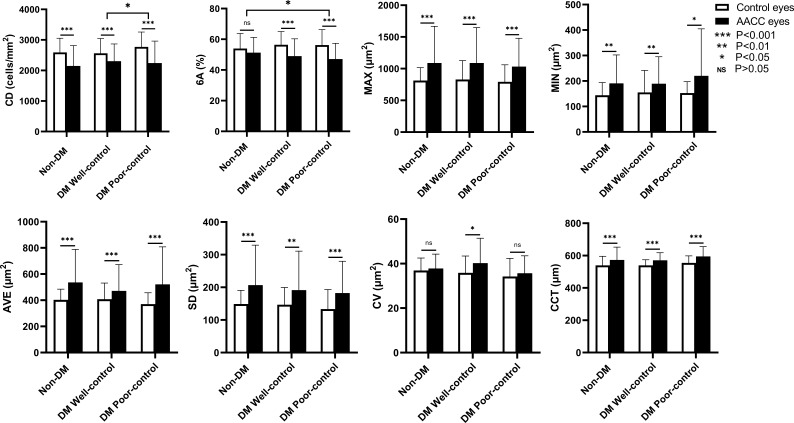
The Corneal Parameters of Acute Angle Closure Crisis (AACC) Eyes and Control Eyes in Study Groups. AACC, acute angle closure crisis; DM, diabetes mellitus; CD, cell density; 6A, percentage of hexagonal cells; MAX, maxmum cell area; MIN, minimun cell area; AVE, average cell area; SD, standard deviation of average area; CV, coefficient of variation of average area; CCT, central corneal thickness. The changes of CD, 6A, MAX, MIN, AVE, SD and CCT in each group were statistically significant. CV, known as the descriptive data, was not statistically significant in non-DM group and DM poor-control group.The CD loss rate between DM well-control group and DM poor-control group was statistically significant. The 6A decline rate between non-DM group and DM poor-control group was statistically significant.

For the non-DM group, central endothelial CD was 2148.58 ± 667.64/mm^2^ in the AACC eyes and 2593.00 ± 460.93/mm^2^ in the control eyes, with a loss rate of approximately 17.1% (*P*<0.001). For the DM well-control group, central endothelial CD was 2304.51 ± 563.55/mm^2^ in the AACC eyes and 2562.92 ± 483.72/mm^2^ in the control eyes, with a loss rate of approximately 10.1% (*P*<0.001). For the DM poor-control group, central endothelial CD was 2241.42 ± 720.21/mm^2^ in the AACC eyes and 2770.77 ± 488.58/mm^2^ in the control eyes, with a loss rate of approximately 19.1% (*P*<0.001). While neither the change in 6A and CV in the non-DM group nor the changes in CV in the DM poor-control group were statistically significant, we detected significant differences in the variations of MAX, MIN, AVE, and SD (*P*<0.05). For the non-DM group, CCT was 573.24 ± 79.07μm in the AACC eyes and 539.55 ± 56.10μm in the control eyes, with an increase rate of approximately 6.1% (*P*<0.001). For the DM well-control group, CCT was 570.67 ± 47.15μm in the AACC eyes and 539.46 ± 35.06μm in the control eyes, with an increase rate of approximately 5.9% (*P*<0.001). For the DM poor-control group, CCT was 595.27 ± 60.24μm in the AACC eyes and 554.67 ± 43.79μm in the control eyes, with an increase rate of approximately 7.2% (*P*<0.001) (**Table 2**; **Figure 1**). However, comparing these corneal parameters of the control eyes revealed no significant differences between the three groups (*P*>0.05).

**Table 2:** Corneal parameters of acute angle closure crisis (AACC) eyes and control eyes in study groups.

Corneal Parameter			CD (/mm^2^)	6A (%)	MAX (μm^2^)	MIN (μm^2^)	AVE (μm^2^)	SD (μm^2^)	CV (μm^2^)	CCT (μm)
Non-DM Group		AACC eyes	2148.58 ± 667.64	51.33 ± 9.93	1089.93 ± 577.38	190.69 ± 111.69	536.66 ± 252.42	206.67 ± 122.52	37.82 ± 6.44	573.24 ± 79.07
		Control eyes	2593.00 ± 460.93	53.99 ± 9.75	813.00 ± 202.46	143.78 ± 50.02	402.60 ± 81.78	148.88 ± 42.03	36.93± 5.57	539.55 ± 56.10
		Test value	t = -5.726	t = -1.516	t = 4.138	t = 3.242	t = 4.453	t = 4.158	t = 0.932	t = 4.043
		P value	**P < 0.001**	P = 0.134	**P < 0.001**	**P = 0.002**	**P < 0.001**	**P < 0.001**	P = 0.355	**P < 0.001**
		Change Rate (%)	17.1	5.6	34.1	32.6	33.3	38.9	2.7	6.1
DM Group	Well-control	AACC eyes	2304.51 ± 563.55	49.03 ± 11.28	1089.97 ± 557.71	189.44 ± 105.88	471.59 ± 200.26	191.62 ± 119.24	40.23±11.20	570.67 ± 47.15
		Control eyes	2562.92 ± 483.72	56.44 ± 8.61	829.41 ± 297.37	155.00 ± 86.47	408.03 ± 122.85	146.97 ± 52.63	35.90 ± 7.51	539.46 ± 35.06
		Test value	t = -6.082	t = -3.797	t = 3.900	t = 3.143	t = 3.640	t = 2.710	t = 2.662	t = 5.165
		P value	**P < 0.001**	**P < 0.001**	**P < 0.001**	**P = 0.003**	**P < 0.001**	**P < 0.01**	**P = 0.011**	**P < 0.001**
		Change Rate (%)	10.1	13.1	31.5	21.9	15.7	30.6	12.0	5.7
	Poor-control	AACC eyes	2241.42 ± 720.21	47.13 ± 10.22	1032.75 ± 447.00	220.40 ± 184.52	521.52 ± 286.10	182.35 ± 97.49	35.69 ± 7.81	595.27 ± 60.24
		Control eyes	2770.77 ± 488.58	56.21 ± 10.06	792.83 ± 267.62	152.56 ± 44.72	370.44 ± 86.69	133.50 ± 59.71	34.23 ± 8.17	554.67 ± 43.79
		Test value	t = -6.018	t = -4.725	t = 3.942	t = 2.377	t = 3.768	t = 3.471	t = 1.025	t = 6.573
		P value	**P < 0.001**	**P < 0.001**	**P < 0.001**	**P = 0.022**	**P < 0.001**	**P < 0.001**	P = 0.311	**P < 0.001**
		Change Rate (%)	19.1	16.2	30.3	43.8	40.7	35.8	4.3	7.2

AACC, acute angle closure crisis; DM, diabetes mellitus; CD, cell density; 6A, percentage of hexagonal cell; MAX, maximum cell area; MIN, minimum cell area; AVE, average cell area; SD, standard deviation of average area; CV, coefficient of variation of average area; CCT, central corneal thickness.

Bold values indicate statistically significant. (P < 0.05).

**Figure 2:**
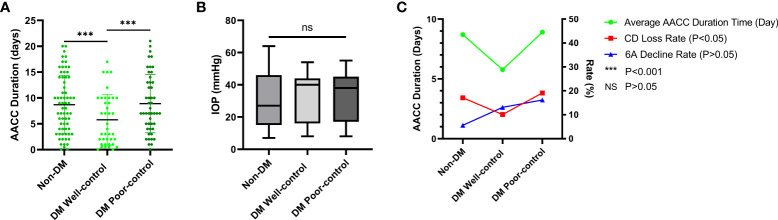
The Average Duration Time of Acute Angle Closure Crisis (AACC), Intraocular Pressure (IOP) of AACC Eyes and Correlations between the AACC Duration, Cell Density (CD) Loss Rate and Percentage of Hexagonal Cell (6A) Decline Rate. DM, diabetes mellitus. **(A)** The scatterplot showed the tendency of duration time of three groups, the line of each group meant the average duration. Statistically significant differences were detected between non-DM group and DM well-control group (P < 0.001), DM well-control group and DM poor-control group (P < 0.001). **(B)** The boxes showed the average IOP of 10-90 percentile, the line in box meant the median of IOP. Both the differences of average IOP and median of IOP were not statistically significant among three groups. **(C)** Line chat showed average AACC duration was correlated with CD loss rate, but not with 6A decline rate.

We also analyzed the corneal parameters change of the AACC eyes and the control eyes between the three groups. Finding the variation in CD loss was statistically significant between the DM well-control and DM poor-control groups (*P*<0.05), the 6A change was statistically significant between the non-DM and DM poor-control groups (*P*<0.05). Contrarily, variations in MAX, MIN, AVE, SD, CV, and CCT showed trends of change, but the differences were not statistically significant (*P*>0.05) (**Figure 1**). Meanwhile, the difference in the average duration times of the AACC eyes was statistically significant intergroup (*P*<0.05) (**Figure 2A**). However, there were no significant intergroup differences in IOP (*P*>0.05) (**Figure 2B**).

**Figure 3:**
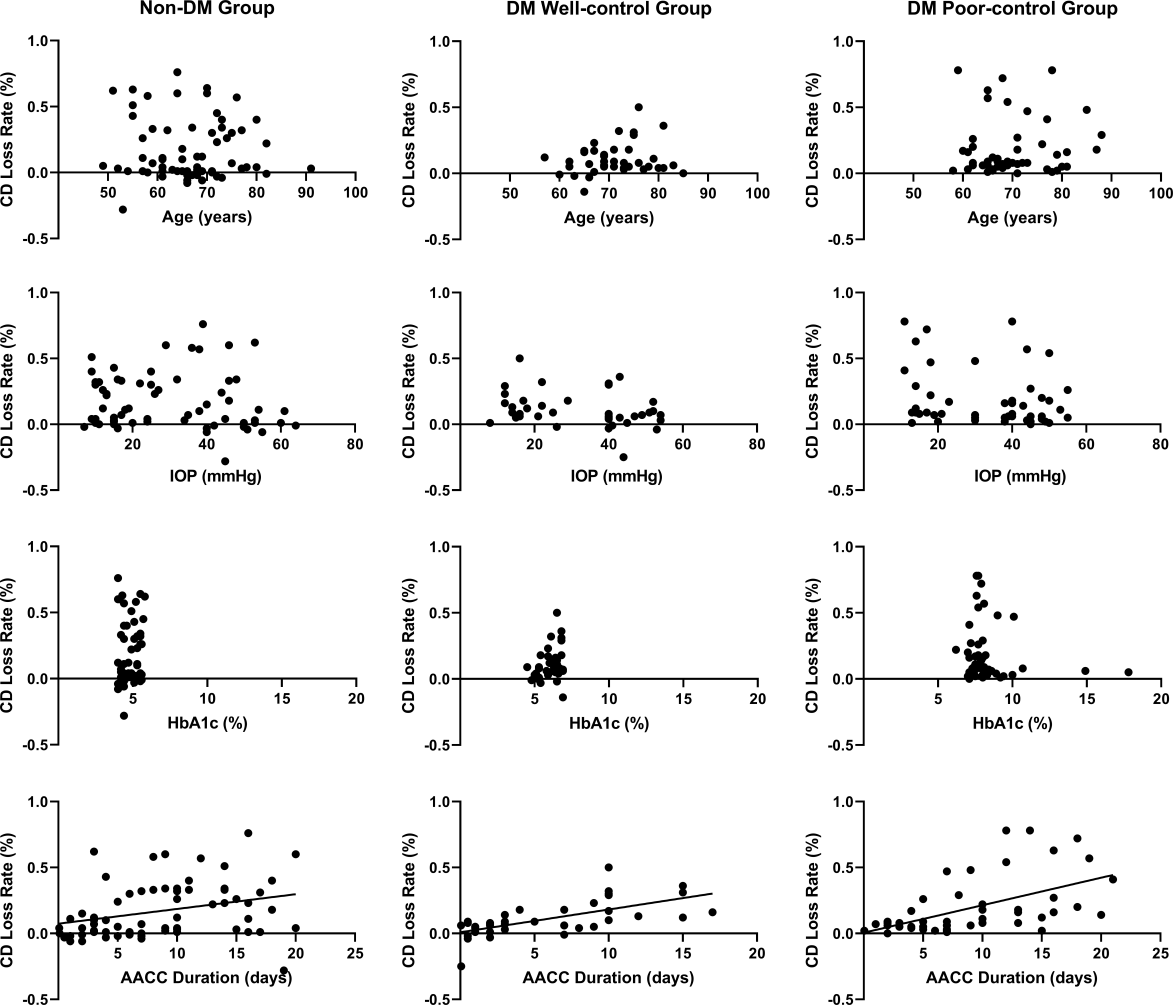
The Relationship between Age, Intraocular Pressure (IOP), Glycosylated Hemoglobin (HbA1c), Acute Angle Closure Crisis (AACC) Duration Time and Cell Density (CD) Loss Rate DM, diabetes mellitus. The AACC duration time was positively linearly correlated with the CD loss rate.

**Figure 4:**
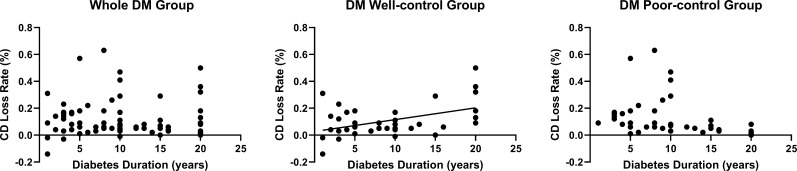
The Relationship between Diabetes Duration Time and Cell Density (CD) Loss Rate in Diabetes Mellitus (DM) Patients. The diabetes duration was positively linearly correlated with the CD loss rate in DM well-control group. 9 patients were excluded from the statistical analysis because they were diagnosed with DM at the time of visit.

Furthermore, we evaluated the correlation of age, IOP, HbA1c, AACC duration, diabetes duration with CD loss rate, and 6A decline rate. None of these data were correlated with the 6A decline rate (*P*>0.05) (**Table 3**). We observed that the 6A decline rate was highest in the DM poor-control group. The comparison between the non-DM group and the DM poor-control group was statistically significant. Importantly, the AACC duration time was correlated with CD loss rate among the three groups (*P*<0.05) (**Figure 2C**). Within each group, there was a linear correlation between the AACC duration and CD loss rate (**Table 3**; **Figure 3**). We also discovered an interesting phenomenon: there was a linear correlation between the diabetes duration time and CD loss rate in the DM well-control group (**Table 3**; **Figure 4**).

**Table 3:** Multivariate regression analysis of the age, intraocular pressure (IOP), HbA1c, AACC duration, diabetes duration and endothelial cell density (CD) loss rate andpercentage of hexagonal cells (6A) decline rate.

CD Loss Rate	Non-DM Group		DM Group			
			Well-control		Poor-control	
	B	P Value	B	P Value	B	P Value
Age	-0.002	0.621	0.004	0.239	0.002	0.722
IOP	-0.001	0.369	-0.003	0.068	-0.003	0.146
HbA1c	0.027	0.623	0.056	0.062	-0.018	0.284
AACC duration	0.011	**0.013**	0.017	**<0.001**	0.021	**<0.001**
Diabetes duration	–	–	0.009	**0.006**	-0.008	0.056
**6A Decline Rate**	**Non-DM Group**		**DM Group**			
			**Well-control**		**Poor-control**	
	**B**	**P Value**	**B**	**P Value**	**B**	**P Value**
Age	-0.0003	0.950	-0.002	0.748	0.006	0.257
IOP	-0.003	0.227	-0.002	0.519	-0.005	0.052
HbA1c	0.017	0.803	0.013	0.825	0.007	0.735
AACC duration	-0.001	0.897	-0.005	0.484	0.001	0.917
Diabetes duration	–	–	-0.0005	0.940	0.010	0.161

IOP, intraocular pressure; HbA1c, glycosylated hemoglobin; AACC, acute angle closure crisis; CD, cell density; 6A, percentage of hexagonal cells; DM, diabetes mellitus; B, unstandardized coefficients.

Bolded values indicate statistical significance.

**Figure 5:**
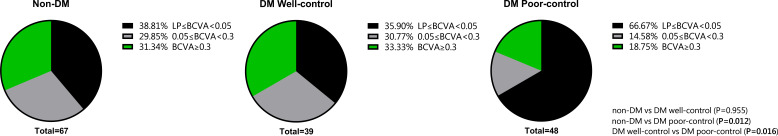
The Best-corrected Visual Acuity (BCVA) Distribution of Acute Angle Closure Crisis (AACC) Eyes in Three Study Group DM, diabetes mellitus. Bolded values indicate statistically significant. The blindness grade of BCVA in DM poor-control patients accounted for 66.67%, which was statistically significant compared to that of the non-DM group and DM well-control group.

Last, we assessed the participants for blindness (LP≤BCVA<0.05), low vision (0.05≤BCVA<0.3), and normal vision (BCVA≥0.3) according to the World Health Organization (WHO) visual impairment and visual acuity criteria. We graded blindness in the AACC eyes for 38.81%, 35.90%, and 66.67% of the participants in the non-DM, DM well-control, and DM poor-control groups, respectively. These differences were also statistically significant (*P*<0.05) (**Table 4**; **Figure 5**).

**Table 4:** The BCVA Distribution of AACC Eyes in Study Groups.

BCVA Grade	Non-DM Group (N)	DM Group (N)	
		Well-control	Poor-control
LP≤BCVA<0.05	26	14	32
0.05≤BCVA<0.3	20	12	7
BCVA≥0.3	21	13	9
Total	67	39	48

BCVA, best-corrected visual acuity; AACC, acute angle closure crisis; DM, diabetes mellitus; N, number.

## Discussion

The corneal endothelium is located on the posterior surface and consists of closely interdigitated hexagonal-shaped cells that are responsible for maintaining corneal transparency. It can both generate fluid secretion by way of an ion transport mechanism and provide an efficient pathway for corneal nutrient uptake and waste removal from aqueous fluid through diffusion and a secondary active transport mechanism (23–25). Multiple exogenous and endogenous factors can cause corneal endothelial cell injury (26). Irreversible vision loss will occur if such an injury is not well controlled. Although many studies have investigated the engineering of human corneal endothelial cells *in vitro*, endothelial damage is still irreversible *in vivo (* 27–29).

In clinical practice, ophthalmologists and scientific researchers are thoroughly aware of the pathogenesis resulting in diabetic retinopathy. Meanwhile, the mechanism of diabetic keratopathy remains unclear. For that reason, diagnosis and treatment have recently become prominent areas of investigation and experimentation. Although many researchers have focused on how type 2 DM affects corneal endothelial status and corneal thickness, their conclusions have largely been inconsistent (30–33). While ophthalmologists understand that phacoemulsification can seriously affect corneal endothelial cells in patients with diabetic cataracts and that the corneal endothelium requires enhanced protection during surgery, there is still insufficient evidence on how rapid IOP fluctuations affect corneal endothelial injuries in glaucoma patients with type 2 DM.

In this study, AACC had different effects on corneal endothelial parameters and CCT in the non-DM, DM well-control, and DM poor-control groups. The central corneal CD and 6A declined while the MAX, MIN, AVE, and SD increased. In the DM group, whether blood glucose was well controlled or not, the 6A decline rate was much more obvious than that in the non-DM group after AACC attack, which supports the previous research (31). In this case, we considered that diabetes could make the morphology of endothelial cells more susceptible to IOP fluctuations. Further, both the variation in CD loss and 6A decline were statistically significant among the groups. We also compared corneal endothelial parameters and CCT in the control eyes among the three groups. While there were some differences, none were statistically significant, thus supporting the previous findings (30). Hence, we concluded that without stimulating rapid IOP fluctuation, there were generally few effects on the corneal parameters or CCT of the control eyes in the three groups, regardless of blood glucose levels. We also detected five-site cell density of the cornea in random patients but found no remarkable correlations between the detection site and injury severity. The CV result was also controversial. Thus, we stipulate that cornea endothelial cells may lie in a sensitive state and are more susceptible to damage under stimulation in patients with DM. Our team preliminarily confirmed that attenuating mitophagy and endoplasmic reticulum stress play important roles in diabetic corneal endothelial dysfunction (34, 35). Nevertheless, continued laboratory research is needed to confirm the exact molecular mechanisms involved.

Contrary to our expectations, the CD loss rate was distinctly lower in the DM well-control group than in the non-DM group. We further analyzed the issue by posing the following question: Do other factors play a more dominant role than glycemic control? We then targeted the unexpected discrepancy in CD loss rates through statistical analyses of age, IOP, HbA1c, AACC duration time, and diabetes duration time in all three groups. We found that the AACC duration was statistically and significantly much shorter in the DM well-control group than in the non-DM and DM poor-control groups. By contrast, there was a negligible difference in duration between the non-DM and DM poor-control groups. Further, the average AACC duration was correlated with the CD loss rate. However, there were no significant intergroup differences in IOP and HbA1c levels. High IOP can affect the endothelium, depending on the persistence of the period.

Thus, DM well-controlled patients will benefit from seeking early treatment at the hospital, thereby shortening the duration time and reducing the severity of endothelial damage (4). Under normal conditions, good glycemic control can mitigate or avoid damage to corneal endothelium. However, in the DM well-control group, the CD loss rate was higher in patients with a longer DM history. This indicates that DM may cause chronic and imperceptible damage in patients even if the blood glucose was under control.

In general, patients with severe vision loss are more likely to seek early hospital admission and accept treatments. Visual acuity was the direct subjective sensation of patients during AACC attack (36), and BCVA could exclude the ametropia error. To clarify the correlation between BCVA and compliance, we conducted a statistical analysis of visual acuity upon admission for all three groups. To our surprise, 66.67% of the DM poor-control group had BCVA below 0.05, which was considerably more than that of the other two groups. Based on this, we speculated that the BCVA level may also be closely related to patient compliance. In other words, the DM well-control group had better compliance and thus tended to seek medical treatment once symptoms appeared when BCVA was not evidently affected. In contrast, the DM poor-control patients had insufficient compliance, failed to control their glucose levels, and paid little attention to any early-stage ocular symptoms. In addition, due to the neurological damage in DM poor-control patients, they may also be insensitive to the stimulation of high IOP levels (37). In this context, they tended to seek medical help only when visual acuity was noticeably affected, leading to irreversible damage. The participants of different age ranges had different degrees of cataract level, so we did not analyze the BCVA of AACC eyes after the IOP was well controlled, which does not have any obvious clinical value.

Some limitations do exist in this study, such as the inability to obtain corneal parameters before AACC in clinical practice. We had to reduce this error by strictly screening the control eyes and enlarging the sample size. The statistical significance has been verified in the subsequent analysis, and we will continue expanding the sample size in the later stage to verify the current conclusion. About the duration time of AACC, we believed that the early discomfort means the eye pressure is higher than normal. This should be the beginning of the corneal endothelial injury. We made a detailed inquiry into the patients’ history and recorded the time from early symptoms to AACC under control, rather than just after AACC. Despite these limitations, this is still the first study to investigate endothelial injury under AACC attack in non-DM, DM well-control and DM poor-control patients.

In conclusion, we did not find any significant differences in corneal endothelial parameters or corneal thickness of the control eyes between participants with and without DM. However, diabetes may impact the functional status of corneal endothelial cells. Once stimulated by acute IOP fluctuations, these cells are more likely to be harmed and induce cell apoptosis. Better glycemic control could mitigate, but not prevent, the effects of diabetes on corneal endothelium function. Quite possibly, this is also the reason why cataract surgery seems to cause more damage to corneal endothelium in diabetic patients. As such, future studies should focus on verifying the relevant molecular mechanism involved. Further, we found that the AACC duration time was significantly correlated with corneal endothelial injury severity. In this regard, type 2 DM patients should make efforts to improve their compliance and seek treatment upon symptom onset. In addition to keeping their glucose levels under control, they should pay close attention to ocular problems such as blurred vision and ophthalmalgia. This may require education on both—general health consciousness for preventing AACC and the need to receive regular eye examinations. Especially during the early stages, active treatment may significantly reduce the severity of damage, thereby decreasing the risk of vision loss.

## Data availability statement

The original contributions presented in the study are included in the article/supplementary material. Further inquiries can be directed to the corresponding author.

## Ethics statement

The studies involving human participants were reviewed and approved by Medical Ethics Committee of Qingdao Eye Hospital of Shandong First Medical University. The patients/participants provided their written informed consent to participate in this study. Written informed consent was obtained from the individual(s) for the publication of any potentially identifiable images or data included in this article.

## Author contributions

XP contributed to conception and design of the study. LC organized the database and wrote the first draft of the manuscript. YX performed the statistical analysis. All authors contributed to manuscript revision, read, and approved the submitted version.

## Funding

National Natural Science Foundation of China (NSFC), 82101094; and Natural Science Foundation of Shandong province (NSFS), ZR2020QH142.

## Conflict of interest

The authors declare that the research was conducted in the absence of any commercial or financial relationships that could be construed as a potential conflict of interest.

## Publisher’s note

All claims expressed in this article are solely those of the authors and do not necessarily represent those of their affiliated organizations, or those of the publisher, the editors and the reviewers. Any product that may be evaluated in this article, or claim that may be made by its manufacturer, is not guaranteed or endorsed by the publisher.
